# Therapeutics of Infancy and Childhood

**Published:** 1896-07

**Authors:** 


THERAPEUTICS OF INFANCY AND CHILDHOOD. By A. JACOBI, M. D., Clinical Professor of the Diseases of Children in the College of Physicians and Surgeons (Columbia University), New York; President of the Association of American Physicians; late President of the New York Academy of Medicine and of the Medical Society of the State of New York. etc. Small 8vo, pp. 518. Philadelphia: J. B. Lippincott Co. 1896.

Much which this book contains has already been presented by the author in essays and monographs which have appeared from time to time. Some of the articles, we judge, have hardly been retouched in this volume.

The feeding of sick children occupies the first chapter of the book. In his methods of infant feeding Dr. Jacobi differs widely from many pediatrists, but he gives a logical reason for the faith that is in him and his wide experience entitles his views to respect. In the preface the writer states that he believes in medicines, hence throughout the book one finds stress laid on the action of drugs beyond what is usual in works on pediatrics. This feature makes the book a valuable supplement to more pretentious volumes which slight the medicinal treatment of disease. The good sense of the author is shown all through the book, noticeably in his remarks on circumcision in the chapter on diseases of the genito-urinary organs.

Though writing on therapeutics, the author enters sufficiently into etiology and diagnosis to make clear the rationale of his treatment. Altogether, the physician who possesses this book will feel that he has an experienced and sensible consultant who does not hesitate to teach by his mistakes as well as by his achievements.

